# Tackling Food Insecurity with ‘Early Help’ Provided by Social Services: A Mixed-Methods Study

**DOI:** 10.3390/nu18213698

**Published:** 2026-11-07

**Authors:** Debra Jones, Paul Clarkson, Anthony Gilfillan, Jeff Burns, Sorrel Burden

**Affiliations:** 1 School of Health Sciences, University of Manchester, Oxford Road, Manchester M13 9PT, UK; 2 Manchester City Council, Town Hall, Manchester M60 2LA, UK; 3 Salford Royal Hospital, Northern Care Alliance NHS Foundation Trust, Salford M6 8HD, UK

**Keywords:** food insecurity, family wellbeing, social care, Manchester City Council, early help, foodbanks

## Abstract

**Background/Objectives**: Food insecurity (FI) is an increasing concern in the UK. Early Help (EH) provided by social services, based on strengths-based conversations with families, can address wider social and economic needs experienced by families living with FI. This study aimed to describe needs identified through EH assessments among families attending foodbanks, the proportion of recorded needs met, and stakeholder experiences. **Methods**: A mixed-methods study was undertaken using 30 months of routinely collected social services data. Regression modelling examined associations between needs identified at the start of EH and the proportion documented as met at the end of support. Semi-structured interviews explored stakeholder experiences using Framework Analysis. **Results**: Records were available for 385 families comprising 1840 individuals (1108 children; 732 adults). Thirty-five categories of need were recorded, with 2646 needs identified (mean 6.9, standard deviation [SD] 3.2 per family). Of these, 2505 (95%) were documented as met, and 301/385 (78%) families had all identified needs recorded as met. In the primary quasi-binomial regression analysis (*n* = 378), longer duration of EH involvement was associated with a higher proportion of initially identified needs recorded as met (β = 0.139, *p* = 0.017). Area-level deprivation was also associated with the outcome (β = −0.179, *p* = 0.049), whereas household size was not (*p* = 0.249). The positive association with duration was supported by a Gaussian sensitivity analysis (*p* = 0.003). Interviews (n = 23) identified three overarching themes: systemic and structural drivers of FI; lived experiences and service engagement; and visions for improvement. **Conclusions**: Participants described practical and emotional support gained from EH. Families attending foodbanks had multiple needs across diverse domains and most needs were documented as met following EH involvement. However, the study design does not establish that EH caused these outcomes.

## 1. Introduction

Food insecurity is defined as the lack of consistent access to sufficient and nutritious food in socially acceptable ways [1]. This is increasingly relevant in the UK, with food inflation recently peaking at 19.2% in March 2023 [2] and the overall price of food and non-alcoholic beverages rising by 25% between January 2022 and 2024, compared with 9% in the previous 10 years [3]. Food-secure households in the UK decreased from 92% in 2020 to 90% in 2023 [4], with 7.2 million people (11%) living in food-insecure households and 2.3 million living in a household that had accessed a foodbank in the previous 12 months [1]. Between 2023 and 2024, the Trussell Trust, a large national food aid provider, distributed 3.12 million emergency food parcels, its highest annual supply to date [1]. Groups most vulnerable to food insecurity include low-income families, unemployed people, those with chronic illness, and single-parent households [5,6]. Food security is shown to be less likely, and access to foodbanks more likely, in households with gross weekly incomes of less than £200 per week, households receiving any income-related benefits, households with a younger head of household (16–24 years), households in the social renting sector, or households with children [4].

Currently in England, the North West has the highest proportion of households (5%) accessing foodbanks within the last 12 months [4]. A survey among residents in the North West of England revealed that one-third of residents face food insecurity, with 19% experiencing severe food scarcity [7]. This is most profound in households with children. Food-related disparities not only jeopardise health and well-being but also significantly contribute to wider health inequalities. Food insecurity has been linked to increased perceived stress, weakened immune function, and a higher prevalence of gastrointestinal symptoms in adults [8]. In the context of maternal and infant health, it is associated with low birth weight and a greater risk of adverse outcomes in infants [9]. Addressing the root causes of poverty is therefore essential to tackling the complex challenges of food insecurity and reducing the health inequalities it perpetuates.

Early Help (EH) is a fundamental component of Children’s Services, delivered nationally through social services. It reflects the statutory responsibility of local authorities to safeguard and support children, young people, and their families. This is achieved through collaborative, multi-agency working that promotes a coordinated, whole-family approach, enabling needs to be identified at an early stage and addressed through timely, proportionate support [10,11]. This supportive approach can help address some of the underlying drivers of poverty that contribute to food insecurity. Agencies working alongside social services receive training in Early Help Assessment (EHA), including partners across healthcare, policing, education, and the voluntary sector. These partner agencies, in collaboration with social services within a given locality, can initiate and register an EHA and are subsequently supported, as appropriate, to respond to identified needs within families. The use of foodbanks serves as a key indicator of food insecurity, often reflecting acute shortages of access to sufficient and nutritious food [12]. Through EHAs and the use of strengths-based conversations [13], families are supported to identify their needs and increase awareness of local services and interventions. These interventions aim to address underlying social challenges across the five domains outlined in the government’s “Every Child Matters” strategy [14], which may otherwise contribute to food insecurity. Support provided to families may include developing practical life skills such as household budgeting, cooking, and shopping, as well as facilitating access to services such as Citizens Advice for guidance on debt management and benefit entitlements.

This study investigates food insecurity focusing on individuals who have accessed a foodbank and received support through EH provided by social services.

## 2. Materials and Methods

### 2.1. Aim

To describe the needs identified among families attending foodbanks who accessed EH, examine factors associated with recorded needs being met, and explore stakeholder experiences of EH and foodbank services.

### 2.2. Objectives

#### 2.2.1. Quantitative Objectives (EH Service Database)

- Describe the proportion of identified needs recorded as met following EH support
- Explore factors associated with the proportion of identified needs recorded as met.

#### 2.2.2. Qualitative Objectives (Semi-Structured Interviews)

- Explore stakeholder perspectives, including service users, individuals who work or volunteer in foodbanks, and EHP (Early Help Practitioner) on the causes of and responses to food insecurity.
- Examine how strengths-based conversations and the EHA contribute to addressing challenges faced by families experiencing food insecurity.

### 2.3. Design

This study used a sequential mixed-methods design incorporating quantitative analysis of routinely collected EH service data and qualitative interviews with service users, EHP and foodbank staff. The quantitative component described the needs identified among families attending foodbanks who accessed EH services and examined factors associated with the proportion of identified needs subsequently recorded as met. The qualitative component explored stakeholder experiences of food insecurity, access to services and the support provided.

The quantitative and qualitative components were planned independently, and the quantitative findings did not inform interview participant selection or the development of the interview topic guide. Neither component was given explicit methodological priority, as both contributed to addressing the study objectives. Integration occurred primarily during interpretation, when quantitative findings were compared with qualitative themes to identify areas of convergence, divergence and additional explanation.

Qualitative data were collected through interviews and analysed using both deductive and inductive frameworks [15,16] to capture the experiences and insights of key stakeholders involved in the delivery and use of the service. Interviews were conducted during May and June 2025. This study followed the Consolidated Criteria for Reporting Qualitative Research (COREQ) [16].

### 2.4. Ethical Considerations

Ethical approval was obtained from University of Manchester Research Ethics Committee and a data management plan was developed for data storage and the handling of identifiable personal data. All interview transcripts were anonymised by removing names and any identifiable information, and all recordings were deleted immediately after transcription. A £35 shopping voucher was provided to service users and foodbank staff as a thank you for their time; no compensation was offered to Manchester City Council (MCC) staff. No images were used.

### 2.5. Participants

Participants were included if they had accessed EH services and attended a foodbank. Participant information was accessed through pseudo-anonymised data from the ‘Liquid Logic Client Database’ and ‘Supporting Families Outcomes at Closure Dataset’. Only data from the previous 30 months, after the COVID-19 pandemic period, were included. Any records that did not reference foodbanks and EH were excluded.

Interview participants were recruited consecutively through MCC employees. Initial contact was made by EHP, who may or may not have had a pre-existing relationship with the participant, who were either service users (SUs), foodbank staff/volunteers (FB), or EHP. Data collection and analysis proceeded in parallel. Sample adequacy was assessed during data collection through ongoing review of the emerging themes and the range of perspectives represented across service users, EHP and foodbank staff. Recruitment ceased when further interviews were not generating substantially new themes or insights.

Foodbank attendance was used to identify families accessing food support and was not considered a validated measure of food insecurity.

### 2.6. Data Collection

The quantitative analysis used routinely collected administrative data from the Liquid Logic Client Database and Supporting Families Outcomes at Closure Dataset. Needs were identified during the initial EH assessment, and EH practitioners determined whether these needs had been met, with their status documented in the service records. For this study, a need was classified as met when it was recorded as such by the practitioner. This represented a practitioner-assessed administrative outcome rather than an independently validated improvement in the family’s circumstances. The research team did not independently verify whether identified needs had been resolved.

During interviews, participants were asked to provide feedback on their experiences of accessing or working within foodbanks or EH services. The interviews were led by one interviewer. Each interview ran for approximately 30–45 min and was conducted using a semi-structured approach guided by a pre-defined topic guide, peer reviewed before use. No other non-participants were present for the interviews. Field notes were kept during each interview to aid follow-up on important concepts and to explore any further information. A summary of the main points was relayed during each interview to confirm accuracy of the points raised. Each interview was conducted once with no repeats required. Interviews were conducted by phone call (*n* = 10), in person (*n* = 4), or via Zoom or Teams (teleconferencing software) (*n* = 9), and audio communications were recorded either through Teams recording functionality or through a handheld audio device. In some instances, the audio recording via Teams software included a visual recording, although these types of recordings were only used to aid transcription and were not used in the analysis. Recordings were later transcribed verbatim and then permanently deleted. All transcripts were anonymised by the removal of names and any identifiable information. Transcripts were checked by one researcher against audio recordings and interview notes but were not reviewed by participants themselves.

### 2.7. Data Analysis

Quantitative data identified from the EH service were analysed and described in terms of family characteristics including family size, duration of EH contact, and reason for case closure.

The family was the unit of analysis, with one observation per family. The primary outcome was the proportion of needs identified at the initial EH assessment that were subsequently recorded as met. This was calculated by dividing the number of initially identified needs recorded as met by the total number of needs initially identified. Needs recorded as met without having been identified at the initial assessment were excluded from the numerator. Seven families with missing outcome information for initially identified needs were excluded, leaving 378 families for the primary analysis. A generalised linear model with a quasi-binomial distribution and logit link was used to examine associations between the proportion of initially identified needs met and area-level deprivation, household size and duration of EH involvement. To account for variation in the number of identified needs between families, the binomial response was specified using the number of initially identified needs met and not met for each family, with the total number of initially identified needs forming the denominator. Duration was included as a continuous variable measured in months. A binomial model was initially fitted, but substantial overdispersion was identified (Pearson dispersion parameter = 4.018); therefore, quasi-binomial regression was used to account for this additional variability. No random effects were included.

A sensitivity analysis was undertaken using the original needs-met index, calculated by dividing the total number of needs recorded as met by the number identified at the initial assessment. This index could exceed 1 because additional needs were sometimes recorded as met without having been identified initially. A Gaussian generalised linear model with an identity link was fitted using the same explanatory variables. Examination of residual plots identified departures from normality and heteroscedasticity; therefore, heteroscedasticity-consistent type 3 (HC3) heteroscedasticity-robust standard errors were used. Regression coefficients, 95% confidence intervals and *p*-values were reported. Statistical significance was set at *p* < 0.05. Analyses were undertaken using Stata (version 14) and R (version 4.5.1).

Qualitative data were managed using NVivo version 15 (QSR International) (NVivo) and analysed using Framework Analysis [15,17]. In alignment with Framework Analysis, the thematic framework was developed both inductively from the themes emerging from the interview transcripts and deductively using the research questions and topic guide [17]. The five-step process of Framework Analysis involves (1) familiarisation, (2) developing a thematic framework, (3) indexing, (4) charting, and (5) data mapping and interpretation. One researcher listened to the recorded interviews to start the process of familiarisation and then read and reread the transcripts to check accuracy and become fully familiar with them. The thematic framework was applied to the interview transcripts and then summarised into charts. The charts were discussed and agreed between two researchers. Finally, data mapping and interpretation were discussed and agreed upon. Where quotations are used, only the participant role (e.g., SU, FB, EHP) is noted to preserve confidentiality.

### 2.8. Integration of Mixed-Methods Findings

The quantitative and qualitative findings were integrated during interpretation using a comparative approach. Findings from the EH service database were considered alongside themes identified through interviews with service users, EH practitioners and foodbank staff. Areas of convergence were identified where experiences described during interviews reflected needs recorded in the administrative data. Divergence was considered where qualitative findings highlighted experiences or continuing difficulties that were not fully represented by the quantitative outcomes.

The qualitative findings provided additional context for interpreting the high proportion of needs recorded as met, particularly where participants described continuing social, financial or structural challenges despite receiving support. Integration was presented using a joint display summarising the quantitative findings, corresponding qualitative themes and their combined interpretation (Supplementary Table S1).

### 2.9. Rigour

Rigour was strengthened using a reflective diary and detailed interview field notes. Additional measures to enhance credibility included regular discussion and consensus between two researchers during charting, data mapping, and interpretation. Researcher positionality also informed the analytical process, with both researchers identifying as female and bringing a combination of clinical and research experience across NHS, community, and acute care settings.

## 3. Results

### 3.1. Early Help Service Database

**Figure 1:**
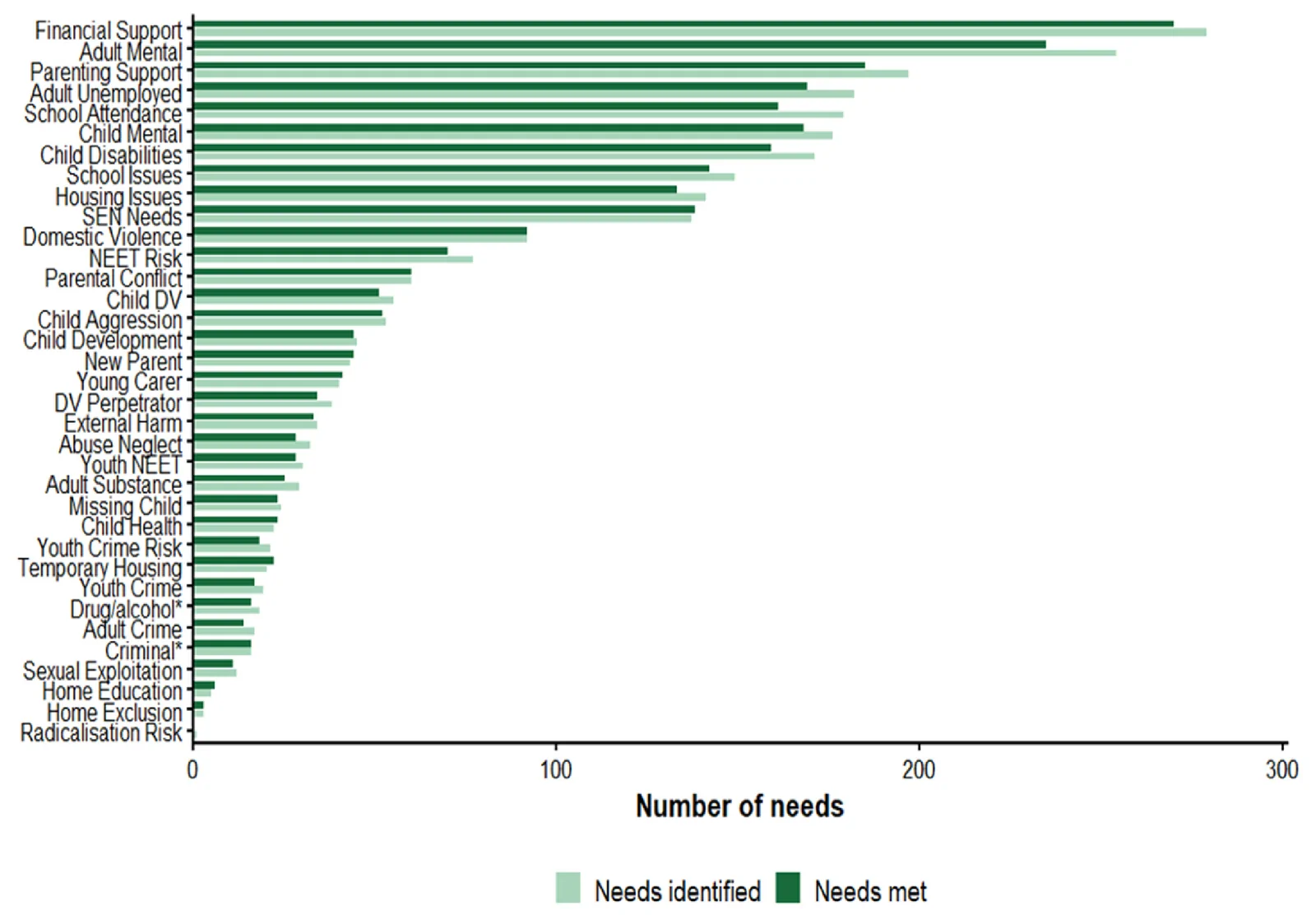
Needs identified and needs met by Early Help intervention. In some domains, the number of needs recorded as met exceeds the number initially identified because additional needs were identified and addressed during Early Help involvement. These additional needs are included in the descriptive figure but were excluded from the numerator of the primary quasi-binomial regression analysis, which considered only needs identified at the initial assessment and subsequently recorded as met. SEN = Special educational needs, NEET = Not in education, employment or training, mental = mental health, Adult substance = adult substance abuse alcohol/drugs, Child substance = child substance abuse alcohol/drugs, DV perpetrator = adult perpetrator of domestic violence, Child DV = child affected by domestic violence, Domestic violence = family affected by interpersonal violence.

The sample comprised 385 families registered for EH who had received food aid from October 2022 to March 2025 over 30 months; the family was the unit of analysis. Families included a mean of 4.78 individuals (SD = 2.3), ranging from 2 to 14 members, and represented a total of 1840 individuals: 1108 children, 585 adults aged 25 years and over, and 147 young adults aged 18–24 years. Most families (305; 80%) lived in areas within the lowest quintiles of the IMD, and the sample covered all areas of Manchester. Thirty-five categories of distinct needs were recorded, with 2646 needs identified (mean 6.9, SD 3.2 per family). Of these, 2505 (95%) were documented as met, and 301/385 (78%) families had all identified needs recorded as met (Figure 1).

**Figure 2:**
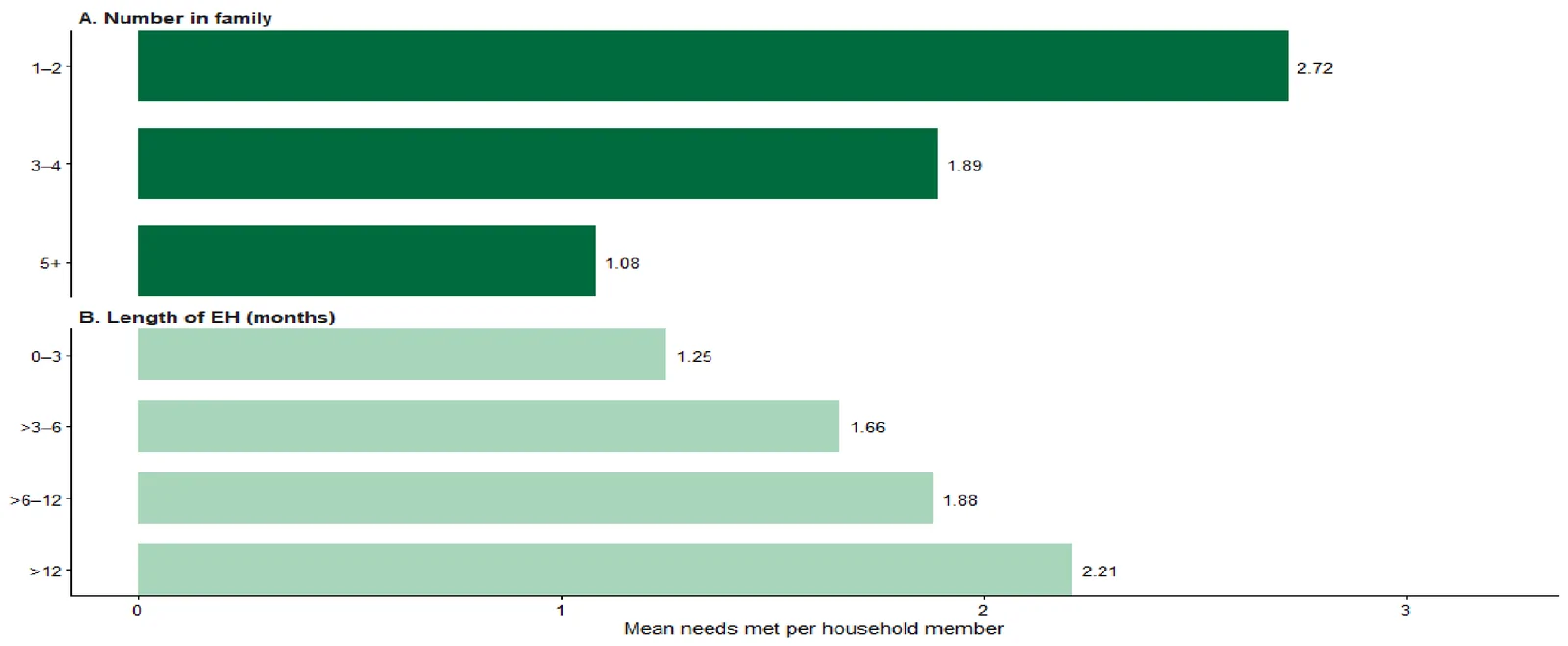
Mean number of needs met per household member according to (**A**) household size and (**B**) duration of Early Help intervention. EH = Early Help.

Families received EH intervention for a median duration of four months (IQR 2–7), with intervention lengths ranging from less than 1 month to 19 months. Thirty-nine families (10.1%) received support for less than one month, 95 (24.7%) for one to three months, 112 (29.1%) for more than three to six months, 118 (30.6%) for more than six to 12 months, and 21 (5.5%) for more than 12 months (Figure 2). Although Figure 2 suggests variation in the number of needs met according to family size, family size was not significantly associated with the proportion of initially identified needs recorded as met in the multivariable analysis.

For most families (341; 89%), EH involvement ended following completion of the intervention. In 11 families (3%), EH was discontinued because support was stepped up to statutory social work involvement. A further 10 families (3%) disengaged, leading to case closure. Other reasons for ending EH included families or children moving out of the catchment area, children becoming looked after, referral to partner agencies, or missing data in a small number of cases.

The mean number of needs identified per family was 6.87, while the mean number of needs recorded as met was 6.51. The mean needs-met index was 0.956, with 301 families (78.2%) having an index of exactly 1 and 27 families (7.0%) having an index greater than 1.

The most frequently identified needs related to financial support, adult mental health support, and parenting support. Table 1 presents the factors associated with the proportion of needs met and provides estimates of the strength of association with service duration and Table 2 shows the sensitivity analysis.

A multivariable quasi-binomial regression analysis was undertaken to examine factors associated with the proportion of initially identified needs subsequently recorded as met. Seven families with missing outcome information were excluded, leaving 378 families in the primary analysis. The model accounted for overdispersion (Pearson dispersion parameter = 4.018) and included area-level deprivation, family size and duration of EH involvement as explanatory variables.

Longer duration of EH involvement was positively associated with the proportion of initially identified needs recorded as met (β = 0.139 per month, 95% CI 0.026 to 0.252, *p* = 0.017). Area-level deprivation was also associated with the outcome (β = −0.179, 95% CI −0.357 to −0.001, *p* = 0.049), although the association was of borderline statistical significance. Family size was not significantly associated with the proportion of needs recorded as met (β = 0.096, 95% CI −0.068 to 0.260, *p* = 0.249) (Table 1).

A sensitivity analysis was undertaken using a Gaussian generalised linear model with HC3 robust standard errors and the original needs-met index, which included additional needs recorded as met without having been identified at the initial assessment. This analysis included all 385 families. Longer duration of EH involvement remained positively associated with the needs-met index (β = 0.012 per month, 95% CI 0.004 to 0.020, *p* = 0.003). Neither area-level deprivation (β = −0.013, 95% CI −0.032 to 0.007, *p* = 0.205) nor family size (β = −0.004, 95% CI −0.014 to 0.007, *p* = 0.469) was significantly associated with the outcome (Table 2).

The primary and sensitivity analyses therefore demonstrated consistent positive associations between duration of EH involvement and recorded needs met. However, the association with area-level deprivation was observed only in the primary quasi-binomial analysis, indicating that this finding was sensitive to the outcome definition and modelling approach.

### 3.2. Interviews

**Figure 3:**
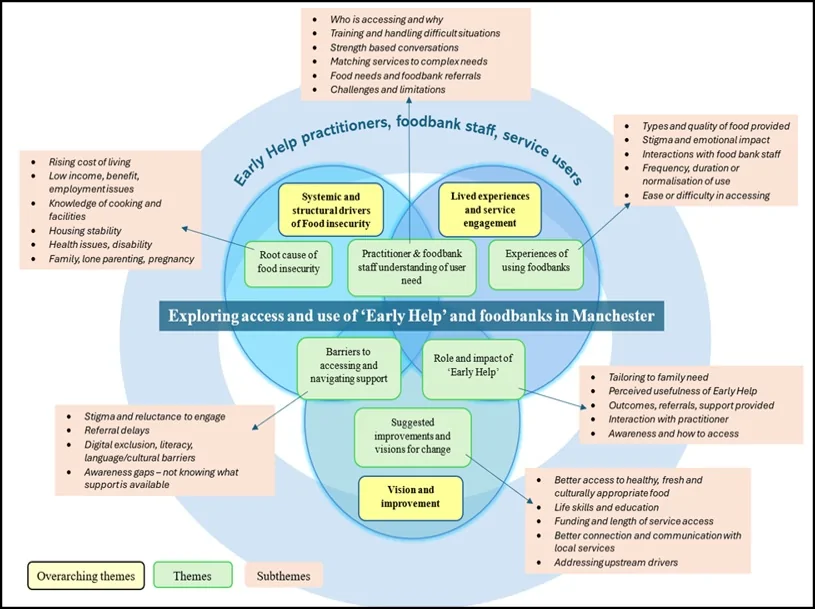
Qualitative overarching themes, themes and sub-themes identified from stakeholders of the Early Help service in Manchester during semi-structured interviews.

Interviews were conducted over May and June 2025, and 23 participants were interviewed out of 37 approached. Participants included 13 SUs, five EHPs, and five FB participants. SUs had a mean (SD) age of 41.8 (8.2) years, and the majority were female. Just over half were White British, with the remainder being White European or other. Most SUs had school-aged children living with them, and just under a quarter had an infant under school age. Half of participants had between 2 and 4 people in the household, with the other families having five or more people in the household. Nearly all were unemployed or on sick leave. Most were also claiming benefits; however, just under a third were unsure of their weekly household income, just under 40% thought their household income was below £200 per week, and the remaining participants thought it was above £200 per week. In terms of cooking facilities and food storage, most had an oven, hob, microwave, and adequate cupboard space; however, just under a third felt that their fridge and freezer were inadequate. EHPs had a mean (SD) age of 44.2 (8.9) years; the majority were female and White British; all were educated to GCSE level and above; and all had at least 3 years’ experience in the role. FB participants had a mean (SD) age of 42.6 (7.6) years, gender split was fairly even, ethnicity was either White British or British Pakistani, and all had at least 1 year of experience in the role. All participants and services were located across North, South and Central Manchester. The qualitative analysis identified three overarching themes encompassing sub-themes (Figure 3). These reflected the structural drivers of food insecurity, experiences of accessing and receiving support, and opportunities for improving services.

### 3.3. Root Causes of Food Insecurity

The rising cost of living was seen by many as a major contributor to food insecurity, making it difficult to afford basic groceries. Service users also highlighted the trade-offs they often had to make, including buying less healthy frozen food instead of fresh food.

‘*To be honest things are getting too expensive at the supermarket and sometimes I don’t have the money to really to do like a big proper shop. So, I often like run low on food stuff plus a 16-year-old son that eats a lot [laughs] trust me*’

(FAM820)

Low income and benefit delays were also an issue, with many families describing being left without income due to delays, errors, or changes in the benefits system, which forced reliance on foodbanks and created ongoing uncertainty.

Despite these issues, many family members stated that they have good cooking skills and enjoy preparing meals from scratch. However, parents often adapted meals, relied on cheaper ingredients, or defaulted to frozen or processed foods.

‘*We have things like burger pasta because mince is too dear…. The kids love it. But it’s a cheap spaghetti Bolognese isn’t it*’

(FAM813)

### 3.4. Practitioner and Foodbank Staff Understanding of User Need

Practitioners and FB participants described the families accessing support services as having a wide range of needs, from mental health and educational concerns to domestic abuse, financial struggles, and housing issues. They stated that families access support through multiple routes, including self-referral, referral from a local service such as schools or police, or through word of mouth.

The general feedback on strengths-based conversations was positive, with EHPs describing them as an effective way to understand families’ strengths and challenges and to put in place an achievable action plan.

‘*Families open up to those questions and then they lend a lot into that. But I think if you don’t go into that kind of the strength questioning, I think you can miss out some stuff*’

(EHP102)

The EH service and food banks offer a coordinated approach for families in immediate crisis who require assistance to access food. Staff stated that referrals to foodbanks, community pantries, and local schemes address immediate hunger but also offer ongoing support and connect families to wider resources. Despite this, practitioners and FB participants reported a number of challenges and limitations to the services offered, with many foodbank and charitable referrals being short term, restricted, or dependent on strict criteria.

### 3.5. Experience of Using Foodbanks

Service users had mixed experiences of the types and quality of food provided by foodbanks, ranging from appreciation for the essentials to frustrations with the poor quality, lack of fresh options and limited choice. However, some participants commented that some foodbanks had fridges and were able to offer fresh foods, which was seen as a positive. However, this was not considered the norm, as most foodbanks offered non-perishable foods.

‘*There’s a standard list of mostly tins and mostly non-perishable food including UHT milk and jam, and tins of veg, tins of fruit, things like that, and you get some toiletries….you get some choice but most of it is allocated for the amount and certain amount of tins per person*’

(FAM803)

Interaction with FB also varied, ranging from friendly and supportive engagement to more procedural or impersonal experiences. These interactions were often described as routine and focused on verifying eligibility and distributing food fairly.

However, many people emphasised that staff were kind, patient and respectful, creating an environment free from judgement. In some cases, participants stated that FB connected them to wider services such as Citizens Advice or community activities.

‘*Erm there is one [foodbank] in [Town], in that church, and they had Citizen’s Advice, a table there. Erm which I actually found that really, really helpful…I needed to go to the food bank but it was like an added bonus…I stayed there for an half an hour that day, but that’s the only one I’ve had like that*’

(FAM821)

In terms of frequency of use, some participants stated that they only turned to foodbanks occasionally, while others used them regularly across multiple sites, with many describing them as an essential lifeline despite limited provision.

### 3.6. Role and Impact of Early Help

Most participants spoke positively about EH, stating that support was highly individualised and responsive to family circumstances. Participants described EHPs going above and beyond to offer emotional support, practical help, and tailored referrals. In general, feedback suggested that participants valued the personal attention, whether through home visits, listening to children, securing essential household items, or connecting them to wider services.

‘*She helps me mentally, physically. Erm, she helps financially at times. She’s—She helps my son, erm, and she…She doesn’t come across as like, as a snob. She’s just really great. Well, she’s great for our—for what my family needs*’

(FAM819)

Importantly, EH was perceived as a positive alternative to more disciplinary interventions, like social services, offering preventative support that kept families engaged and reduced stress.

‘*So I think with the right support in place and I do think Early Help are a main source of that really, before throwing social services in straight away, because it could have gone a lot different for me and my partner, you know what I mean?*’

(FAM813)

Families consistently linked the success of EH to the quality of their relationship with the practitioner. These relationships fostered trust and openness, allowing underlying issues to surface.

‘*She’s so easy to talk to—I don’t like talking to strangers me but [EHP], she proper got me. She was dead down to earth…It’s nice to know that somebody actually knows and understands what you’re going through…I really do think, if I was to give anyone a medal, I think [EHP] would deserve it because she did a lot for me*’

(FAM813)

In terms of awareness, families learnt about EH in different ways, but mainly through having a referral from a local service such as schools or the police. While a few knew of the service, several admitted that they had not been aware that EH even existed until another service connected them. The remaining sub-themes, including suggested changes or improvements and perceived barriers to accessing and navigating support, are in Supplementary Tables S1 and S2.

### 3.7. Integration of Mixed Methods Results

The qualitative findings provided context for the quantitative service records. Financial support, parenting support, and adult mental health were among the most frequently recorded needs within the EH database, and these same issues emerged consistently across interviews as major contributors to food insecurity. Participants described benefit delays, housing insecurity, poor mental health, and parenting pressures as interconnected challenges requiring multiple forms of support. Similarly, while quantitative records documented a high proportion of identified needs as met, interview participants described the types of support received, including emotional support, practical assistance, and referral to wider services, illustrating how these recorded outcomes were experienced in practice. However, qualitative data also highlighted continuing structural barriers, indicating that recorded needs being met did not always equate to resolution of all needs identified within families. This highlights some of the complexities within families that require sustained support. See Table S3: Integration of quantitative and qualitative findings.

## 4. Discussion

This study adds to the limited literature examining the experiences of families accessing both foodbanks and EH services.

### 4.1. Early Help Service Database

The EH database demonstrated that a wide range of needs were identified and that many of these were recorded as met during the period of EH involvement. The qualitative findings help explain how practitioners and families understood these recorded service responses and illustrate the types of support provided. However, because food security was not measured before or after intervention, these findings should not be interpreted as evidence that EH improved food insecurity. There were several points that resonated between the data from social service records and the interviews with recipients of food aid, providers and EHP. The areas of need most identified by families were finances, adult mental health and parenting support. This aligns with previous evidence and surveys carried out recently in the UK, including a survey of 5000 UK adults commissioned by the Mental Health Foundation in 2023, which found that 58% of people with young children had taken on or increased unsecured debt to pay for essential bills, and 32% of these were very worried about being able to repay [18]. A survey of 3564 adults by UNICEF UK in 2022 found that 59% of parents with children under 5 were struggling with mental health and 66% said they had been affected by the rising cost of living [19]. This survey also stated that families were being forced to make impossible decisions due to rising costs of living, with 1 in 5 parents having low incomes and stating that they skip meals to pay for childcare [19].

Longer duration of EH involvement was associated with a greater proportion of initially identified needs being recorded as met in the primary quasi-binomial analysis, and this finding was supported by the Gaussian sensitivity analysis. This association may reflect greater opportunities to address identified needs over time or differences in family circumstances, complexity of needs and service delivery processes. Area-level deprivation was associated with the proportion of initially identified needs met in the primary analysis, although this association was not observed in the sensitivity analysis using the original needs-met index. The findings relating to deprivation should therefore be interpreted cautiously, particularly given the borderline statistical significance and sensitivity to the outcome definition. As these analyses used observational service data, causal relationships cannot be established. The association with duration should not be interpreted as evidence that longer involvement causes better outcomes, as the length of EH support may also reflect family circumstances, complexity of needs and service delivery processes.

Household size was not significantly associated with the proportion of initially identified needs recorded as met in either the primary quasi-binomial regression analysis or the Gaussian sensitivity analysis. However, previous research suggests that larger families may experience greater financial pressures. A survey of 4000 UK households on low incomes conducted between 2022 and 2025 reported that families with three or more children were more likely than smaller families to go without essentials, be behind on bills, and have a loan to pay for essentials. These findings highlight the broader financial challenges experienced by larger families, although such differences were not demonstrated in relation to recorded needs met in the present study [20].

### 4.2. Interviews

Our qualitative data highlighted the critical role that EH and foodbanks play in mitigating immediate hardship, while also underscoring the complex, overlapping challenges that underpin a family’s reliance on support. This is similar to the findings of a systematic review, which concluded that food aid has a crucial role in supporting families and is associated with improvements in food security and diet quality [21].

Families relying on foodbanks faced overlapping pressures, including lone parenting, pregnancy, health and disability challenges, unstable housing, low income, and benefit-related complications. This has also been highlighted in an ethnographic study, which points out that food poverty is embedded in contemporary lived experiences characterised by precarious incomes and insecure housing [22]. These factors combined to limit families’ ability to provide sufficient, nutritious food at home. Housing instability, lack of adequate cooking facilities, food cost and choice, and feeding children were highlighted as additional barriers to preparing food and contributed to reliance on ready-made or processed foods. These findings align with wider UK evidence that food insecurity is best understood as a systemic issue rather than the result of individual choice or poor budgeting [6,23]. Participants often described foodbank use as temporary, triggered by specific circumstances such as benefit delays or health crises, yet these disruptions could have lasting effects [24,25]. Thus, foodbanks act as a crucial safety net, providing immediate relief when necessary.

Awareness of, and access to, EH was often dependent on referrals from schools or other local services. Feedback from families highlighted that engagement with EH was most effective when practitioners demonstrated empathy, built trust, and provided tailored responses that were sensitive to family circumstances. The use of a strengths-based approach within social care promotes a more balanced relationship between practitioners and family members under the umbrella of social justice and empowerment [26]. However, it has been pointed out that a strengths-based approach places the emphasis on individual responsibility, possibly out of context of wider societal issues, including ethnicity, inequality, and poverty [27].

Staff training and strengths-based conversations emerged as critical for positive engagement with families in the present study. Services using a strengths-based approach, compared with traditional approaches, have been associated with positive outcomes for families, including an increased likelihood of gaining employment, increased financial support from social services, improved tolerance for life stresses, and decreased use of secondary healthcare services [27]. Practitioners in the present study described successful interactions with family members as those that recognised hidden needs, connected families to appropriate resources, and balanced immediate relief with longer-term empowerment. This reflects the practical application of the theoretical principles underpinning a strengths-based approach, which moves away from deficit-focused models towards problem-solving within families while considering the broader context of their environment [27].

In terms of foodbanks, participants were generally grateful to be able to access this service, and, in many cases, it served as an essential lifeline, providing food when there was no other alternative. It has been argued that food banks are becoming the “unofficial arm of the welfare state” [28] within society, with basic family needs that are not met by government agencies being met by charitable support. Foodbanks can serve as ‘safe spaces’ where respect, compassion, and personalised engagement support emotional wellbeing and reduce stigma. For some families, food banks also acted as a gateway to additional services, providing opportunities to be signposted to support from other agencies. This highlights how foodbanks can function not only as providers of food but also as spaces where social support and guidance can be accessed.

### 4.3. Strengths and Limitations

A key strength of this study was the use of a mixed-methods approach, combining routinely collected EH service data with interviews involving service users, EH practitioners and foodbank staff. This enabled the quantitative findings to be considered alongside the experiences of families and those providing support, offering a broader understanding of the needs identified and how these were addressed in practice.

However, several limitations should be acknowledged. The quantitative analysis relied on routinely collected administrative data, with the outcome reflecting the proportion of initially identified needs recorded as met within the EH database rather than independently validated improvements in family circumstances or wellbeing. Recording practices, including procedures associated with case closure, may have influenced the high proportion of needs documented as met. Furthermore, food insecurity was not directly measured using a validated instrument, and no baseline or follow-up assessment of food insecurity or comparison group was available. Consequently, the study cannot establish whether EH improved food security or determine the effectiveness of the intervention.

The primary outcome was highly concentrated at the upper boundary, with a substantial proportion of families having all initially identified needs recorded as met. A quasi-binomial regression model was therefore used to accommodate the proportional outcome and account for overdispersion. Although this approach was more appropriate for the outcome distribution, it did not address potential administrative misclassification or the limited variability in recorded outcomes. A Gaussian sensitivity analysis was also undertaken using the original needs-met index, which included additional needs recorded as met without having been identified at the initial assessment. Both analyses demonstrated a positive association between duration of EH involvement and recorded needs met. However, the association with deprivation was observed only in the primary quasi-binomial analysis, indicating that this finding was sensitive to the outcome definition and modelling approach. Residual confounding is also possible, as factors such as the complexity of family needs and intensity of support were not included in the regression models. Furthermore, longer involvement may provide more opportunities for needs to be addressed and recorded as met. These associations should therefore not be interpreted as evidence of causality.

The study included only families who had accessed both foodbanks and EH services. These families may differ from other foodbank users in their circumstances, identified needs, referral pathways and engagement with support services. Selection into EH may therefore have influenced the characteristics of the sample, and the findings should not be considered representative of all families attending foodbanks. Recruitment of interview participants through Manchester City Council employees may also have introduced selection bias, as individuals who remained engaged with services or had more positive experiences may have been more likely to participate. Social desirability bias is also possible, particularly where participants were discussing services from which they had received support. These factors may have influenced the range of experiences represented in the qualitative findings.

Finally, the study was undertaken in Manchester, and the findings may not be directly transferable to other areas with different populations, levels of deprivation or arrangements for delivering EH and foodbank services.

## 5. Conclusions

This mixed-methods service evaluation describes the needs identified among families attending foodbanks who accessed EH, the proportion of those needs recorded as met within routine service records, and stakeholder experiences of the support received. The qualitative findings suggest that families and practitioners valued EH for providing practical assistance, emotional support and connections to wider services, while also highlighting the continuing influence of wider structural drivers such as poverty, housing instability and welfare insecurity. Although many identified needs were recorded as met within the EH database, this represents an administrative assessment of service outcomes and does not necessarily indicate sustained improvements in the underlying social, financial or health-related circumstances of families. These findings should therefore not be interpreted as evidence of intervention effectiveness. Future research should incorporate validated measures of food insecurity collected before and after intervention, alongside appropriate comparison groups, to determine whether EH contributes to sustained improvements in food security and related outcomes.

## Figures and Tables

**Table 1:** Factors associated with the proportion of initially identified needs met following Early Help involvement: quasi-binomial regression analysis (n = 378).

Variable	Beta	SE	95% CI	*p* Value
IMD	−0.179	0.091	−0.357, −0.001	0.049
Family size	0.096	0.083	−0.068, 0.260	0.249
Duration EH (months)	0.139	0.058	0.026, 0.252	0.017

β coefficients are reported on the log-odds scale and represent the change in the log-odds of an initially identified need being recorded as met for each one-unit increase in the explanatory variable. Duration of Early Help involvement was modelled continuously in months. EH = Early Help; IMD = Index of Multiple Deprivation; SE = standard error; CI = confidence interval.

**Table 2:** Factors associated with the needs-met index following Early Help Support (n = 385).

Variable	Beta	SE	95% CI	*p* Value
IMD	−0.013	0.01	−0.032, 0.007	0.205
Family size	−0.004	0.005	−0.014, 0.007	0.469
Duration EH (months)	0.012	0.004	0.004, 0.020	0.003

β coefficients represent the absolute change in the needs-met index associated with a one-unit increase in the explanatory variable. Duration of Early Help involvement was modelled continuously in months. HC3 heteroscedasticity-robust standard errors were used. EH = Early Help; IMD = Index of Multiple Deprivation; SE = standard error; CI = confidence interval.

## Data Availability

Data may be available from the corresponding author upon reasonable request, subject to approval by Manchester City Council’s legal team.

## Supplementary Materials

The following supporting information can be downloaded at: https://www.mdpi.com/article/10.3390/nu18213698/s1, Table S1: Summary of recommended improvements and barriers to accessing services according to service users, food bank staff, and EHPs; Table S2: Summary of barriers to accessing and navigating support through both Early Help and food banks. Table S3: Integration of quantitative and qualitative findings.

## Abbreviations

The following abbreviations are used in this manuscript:

CI	confidence interval
COREQ	Consolidated Criteria for Reporting Qualitative Research
EH	Early Help
EHP	Early Help practitioner(s)
EHA	Early Help Assessment
FB	foodbank staff/volunteers
FI	food insecurity
IMD	Index of Multiple Deprivation
IQR	interquartile range
SD	standard deviation
SE	standard error
SU	service user(s)
