# Supplementary material for: Tackling Food Insecurity with ‘Early Help’ Provided by Social Services: A Mixed-Methods Study
Source: Nutrients. 2026 Nov 7;18(21):3698. doi: 10.3390/nu18213698 (PMC13765437; doi:10.3390/nu18213698)
Supplement: Supplementary file 1 [file nutrients-18-03698-s001.zip › nutrients-4628758-supplementary.pdf]

Supplementary material

Supplementary Table S1: Summary of recommended improvements and barriers to accessing services according to service users, food bank staff, and EHPs.

Suggested changes or improvements	
	Change/improvement
Service users	• Better communication/listening in relation to benefit issues<br>• Better accessibility to food banks (times/locations/delivery)<br>• Easier access to food that doesn't require a referral or voucher<br>• More time to access EH services<br>• More variety at food banks (more fresh or frozen produce as tinned goods not always suitable for kids)
EHP	• Address upstream drivers in relation to benefit and housing issues<br>• Better connection with mental health services and support for young carers<br>• More information on, and better connection with, food banks to know which to refer to and when<br>• Providing more educational material/messages around finances, budgeting and how food fits into that<br>• Add finance and life skills to school curriculum<br>• Increase awareness of EH in the general population through posters and advertisements in local community centres, food banks, online, etc.
Food bank staff	• Addressing upstream drivers in relation to money and benefits so people do not have to rely on food banks<br>• Potential for government to direct money where it is needed: the last cost-of-living payment was related to reduced use of food banks<br>• More opportunities for EH to link in with food banks by attending meetings where local food banks meet as a group<br>• Better communication with large supermarkets/suppliers on working together with surplus food<br>• More funding needed in the short term to keep supporting people and help move them out of crisis

EH: Early Help; EHP: Early Help practitioner(s).

<u>Supplementary Table S2: Summary of barriers to accessing and navigating support through both Early Help and food banks.</u>

**Perceived barriers for accessing and navigating support**	
	**Barriers**
**EH services**	• Stigma around asking for help in terms of food poverty<br>• Cultural gaps where families who need support won't ask for help due to stigma or shame<br>• Working families who need support can be missed as they won't present as having issues and can potentially be earning less than the benefit amount but will be just above threshold to claim<br>• Difficulties in meeting with working families as they don't have the time<br>• Families not aware that they can self-refer and therefore unsure how to engage and stigma around asking for a referral from local services.<br>• Lack of awareness by the public about EH and how to access it<br>• Reluctance to engage as EH is part of children services and there appears to be a worry around asking for help or saying they have no food and what that could lead to in terms of child custody.<br>• Timing may be inappropriate, and families may not be ready to have EH come into their homes<br>• Stigma of a council worker attending a home and how this may be perceived by others.<br>• Worry of single parents and the stigma around being alone and why that might be
**Food bank support**	• Cultural barriers relating to types of food available<br>• Transportation and mobility issues can prevent access<br>• Barrier to attending as food parcel considered to not be worth the cost of getting to a food bank plus cost of a voucher to access food<br>• Difficulties acquiring vouchers and referrals and a lack of understanding around why a referral is needed<br>• Stigma and shame of being seen to be attending a food bank<br>• Reluctance to talk and ask for additional help due to the shame of being in a food bank<br>• Most people do not want to attend a food bank and want to fend for themselves; attending a food bank is often a last resort and means food banks are constantly dealing with reluctance, desperation, and people in crisis
**Other support services**	• Individuals may be prevented from accessing services due to digital exclusion, including not having a phone/laptop or access to internet<br>• Individuals and families may be sanctioned and prevented from accessing money due to 1) mental health/health issues and being unable to follow a process, 2) digital exclusion and incomplete online forms and journals, 3) language / cultural barriers and a lack of understanding around what is required

EH: Early Help

Supplementary Table S3. Integration of quantitative and qualitative findings

Quantitative finding (n = 385 families)	Qualitative finding (n = 23 interviews)	Convergence or divergence	Interpretation
Financial needs were among the most frequently identified needs.	Families described financial hardship, rising food costs, benefit delays and difficulties meeting essential household expenses.	Convergence	Both datasets identified financial insecurity as a major challenge for families accessing food banks and EH.
Adult mental health and parenting support were among the most frequently identified needs.	Service users and practitioners described emotional distress, parenting pressures and the interaction between financial difficulties and family wellbeing.	Convergence	The interviews supported the quantitative findings and illustrated how different family needs were interconnected.
2,505 of 2,646 needs (94%) were recorded as met, and 301 families (78%) had a needs-met index of exactly 1.	Families described practical assistance, emotional support and referrals to other services. However, some continued to experience financial hardship and other structural difficulties.	Partial convergence	The interviews provided examples of support received but also highlighted that needs recorded as met did not necessarily indicate complete or sustained resolution of underlying problems.
Longer duration of EH involvement was positively associated with the needs-met index ($\beta = 0.000394$ per day, $p = 0.003$).	Participants described the importance of developing trusting relationships with practitioners and receiving support tailored to their circumstances.	Complementarity	The qualitative findings suggest possible ways in which ongoing engagement may support families, although they do not establish why longer duration was associated with more needs being recorded as met.
Deprivation and family size were not significantly associated with the needs-met index in the primary regression analysis ($p = 0.205$ and $p = 0.469$, respectively).	Families described financial pressures, difficulties providing food for children and challenges associated with meeting multiple household needs.	Complementarity	The interviews highlighted difficulties experienced by families that were not fully captured by the quantitative associations. The absence of statistically significant associations does not imply an absence of hardship.
341 families (89%) had their EH involvement closed following completion of the intervention.	Families generally valued the individualised support provided by practitioners, particularly practical assistance, empathy and	Partial convergence	Positive experiences of EH were consistent with the high proportion of cases recorded as completed, although administrative case completion cannot be assumed to indicate

	help accessing other services.		satisfaction or sustained improvements.

EH = Early Help. Convergence indicates agreement between quantitative and qualitative findings; complementarity indicates that the qualitative findings provide additional context; partial convergence indicates agreement alongside important qualifications.
